# The Diagnosis of Autism Spectrum Disorder Based on the Random Neural Network Cluster

**DOI:** 10.3389/fnhum.2018.00257

**Published:** 2018-06-26

**Authors:** Xia-an Bi, Yingchao Liu, Qin Jiang, Qing Shu, Qi Sun, Jianhua Dai

**Affiliations:** College of Information Science and Engineering, Hunan Normal University, Changsha, China

**Keywords:** fMRI, random elman neural network cluster, autism spectrum disorder, neural network, classification

## Abstract

As the autism spectrum disorder (ASD) is highly heritable, pervasive and prevalent, the clinical diagnosis of ASD is vital. In the existing literature, a single neural network (NN) is generally used to classify ASD patients from typical controls (TC) based on functional MRI data and the accuracy is not very high. Thus, the new method named as the random NN cluster, which consists of multiple NNs was proposed to classify ASD patients and TC in this article. Fifty ASD patients and 42 TC were selected from autism brain imaging data exchange (ABIDE) database. First, five different NNs were applied to build five types of random NN clusters. Second, the accuracies of the five types of random NN clusters were compared to select the highest one. The random Elman NN cluster had the highest accuracy, thus Elman NN was selected as the best base classifier. Then, we used the significant features between ASD patients and TC to find out abnormal brain regions which include the supplementary motor area, the median cingulate and paracingulate gyri, the fusiform gyrus (FG) and the insula (INS). The proposed method provides a new perspective to improve classification performance and it is meaningful for the diagnosis of ASD.

## Introduction

Autism spectrum disorder (ASD) characterized by impairments in social deficits and communication (Knaus et al., 2008) is a typically neurological disease with high heredity (Baird et al., 2006) and prevalence (Chakrabarti and Fombonne, 2001). It is reported that the prevalence of ASD has increased from 0.67% in 2000 to 1.47% in 2010 (Xu et al., 2018). Thus, the early diagnosis of ASD is meaningful. However, the traditional diagnostic methods are mainly based on clinical interviews and behavior observation, which makes the diagnosis inaccurate. There are two ways that could be applied to improve the diagnostic accuracy of ASD. One of the ways is the usage of the neuroimaging technique, such as Electroencephalogram (EEG; Peters et al., 2013), positron emission tomography (PET; Pagani et al., 2017), structural magnetic resonance imaging (sMRI; Sato et al., 2013) and functional magnetic resonance imaging (fMRI; Ren et al., 2014). The specific properties of fMRI make it widely used (Bennett et al., 2017). Another way is the usage of machine learning which could automatically improve the algorithm performance based on the previous experiences (Jordan and Mitchell, 2015). The neural network (NN) belongs to a branch of machine learning, which is inspired by human brain and has the function of effective pattern recognition. The NN has been successfully employed in the automated classification related to ASD. For instance, Iidaka (2015) applied probabilistic neural network (PNN) to classify ASD patients and typical controls (TC), and the accuracy is close to 90%. Guo et al. (2017) proposed a new feature extraction based on the deep neural network (DNN) to classify the ASD patients and TC, and the accuracy is 86.36%. Heinsfeld et al. (2018) used the deep learning to diagnose ASD, and the accuracy is 70%. Heinsfeld et al. (2018) adopted deep learning to classify the ASD patients and TC, and the accuracy is 70%. These studies fully show that the accuracy of a single NN is not high and unstable in the diagnosis of some diseases.

As the single NN has the advantages of dealing with the imperfect data and solving the problem of complex nonlinear systems, the combination of multiple NNs would combine their differences and also improve classification performance. Therefore, we combine multiple NNs into a model which is named as the random NN cluster in this article. The new method could achieve better feature extraction and classification performance. Specifically, five different NNs are applied to build five types of random NN clusters which are able to classify ASD patients and TC. Then, we compare the accuracies of the five types of random NN clusters. The random Elman NN cluster has the highest accuracy which is approximately close to 100%, thus the Elman NN is selected as the best base classifier. Next, the random Elman NN cluster is used to find the significant features which are able to reflect the difference between ASD patients and TC. Finally, the abnormal brain regions are found out, including the supplementary motor area, the median cingulate and paracingulate gyri, the fusiform gyrus (FG) and the insula (INS). In conclusion, the random NN cluster is an effective method for classification and it could provide a new perspective to improve classification performance in the diagnosis of ASD.

## Materials and Methods

### Demographic Information

In this article, the original imaging data was selected from autism brain imaging data exchange (ABIDE) database^1^), which includes the neuroimaging data of ASD patients and TC. The ASD patients should meet the criteria of childhood autism and the TC should meet the criteria of healthy control. The fMRI data was acquired on 3.0-T Siemens MRI scanner. The sequence parameters include: TR = 3000 ms, TE = 28 ms, matrix = 64 * 64, slice thickness = 0.0 mm, pixel spacing *X* = 3.0 mm, pixel spacing *Y* = 3.0 mm, flip angle = 90°, no slice gap, axial slices = 34, time points = 120. In the scanning process, all participants are expected to lie still and stay awake. Finally, 92 participants that consist of 50 ASD patients and 42 TC were selected out in this article.

The differences of sex and age between the ASD group and TC group were tested by chi-square test and examined by two-sample *t*-test respectively. The results are shown in Table 1. It is referred that there are two groups which have no statistical significance between the sex and the age.

**Table 1:** Basic information of ASD and TC.

Variables (Mean ± SD)	ASD (*n* = 50)	TC (*n* = 42)	*P* value
Sex (M/F)	5/45	6/36	0.528
Age (years)	13.34 ± 2.41	13.05 ± 1.82	0.520

*Abbreviations: ASD, autism spectrum disorder; TC, typical controls*.

### Data Preprocessing

In order to lower the signal-to-noise ratio of fMRI images, all fMRI images need to be preprocessed. In this article, we used the Data Processing Assistant for Resting-State fMRI (DPARSF^2^) software (Chao-Gan and Yu-Feng, 2010). The data preprocessing includes the following eight steps: (a) converting DICOM format to NIFTI format; (b) removing the first 10 time points; (c) slicing timing (Kiebel et al., 2007); (d) realigning with the aim of reducing head motion (Grootoonk et al., 2000); (e) normalizing (Misaki et al., 2010); (f) smoothing with the aim of removing the noise caused by breathing, heartbeat and high frequency signal (Challis et al., 2015); (g) temporal filtering with the aim of regressing out movement vectors by high-pass temporal filtering (Kasper et al., 2014); and (h) removing covariates, such as the whole brain signal, white matter, cerebrospinal fluid signal regression treatment (Lund and Hanson, 2001).

### Basic Theory of the Neural Network

The operation of the human brain always attracts many researchers’ attention. The artificial neural network (ANN) is evolved from the human brain which could achieve an effective nonlinear mapping function. In addition, it has excellent classification performance in different fields such as the field of medicine (Beheshti et al., 2014), economics (Wang et al., 2015) and business (Tkác and Verner, 2016). The following introduces five types of NNs.

#### Backpropagation Neural Network

The Backpropagation (BP) NN is the core of the forward NN and it is able to realize the non-linear mapping (Ren et al., 2014). However, there is no effective method to determine parameters of BP NN, and the network could not be repeated because the initial weights are random numbers.

**Figure 1:**
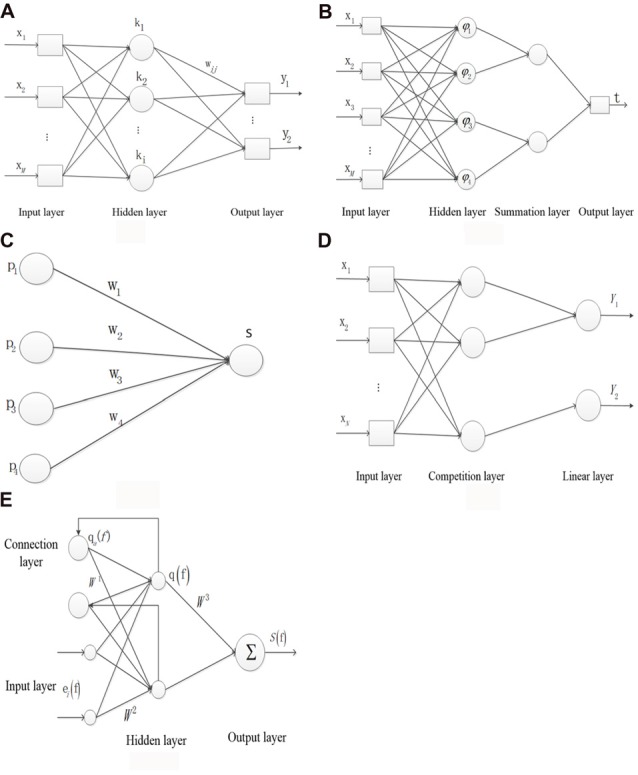
The structure of the five types of neural networks (NNs). **(A)** Backpropagtion neural network. **(B)** Probabilistic neural network. **(C)** Competition neural network. **(D)** Learning vector quantization neural network. **(E)** Elman neural network.

Figure 1A shows the structure of the BP NN. *x* represents the neuron of the input layer, the *mth* output $t_{M}^{m}(n)$ is defined as *x*(*n*) in the input layer, where *n* is the number of iteration and *M* is the number of total inputs. *k* represents the neuron of the hidden layer. *y* represents the neuron of the output layer. *w*_mi_ represents the weight from the *mth* input layer to the *ith* hidden layer, and *w*_ij_ represents the weight from the *ith* hidden layer to the *jth* output layer. *c*(*n*) represents the target output. The *ith* input $s_{I}^{i}(n)$ is denoted as $\sum_{m=1}^{M}w_{mi}(n)t_{M}^{m}(n)$ in the hidden layer, where *I* is the number of neurons in the hidden layer. The output $t_{I}^{i}(n)$ is denoted as $f\left(s_{I}^{i}(n)\right)$ in the hidden layer, where *f*(·) represents the sigmoid function. The *jth* output $t_{J}^{j}(n)$ is denoted as $g\left(\left(s_{J}^{j}(n)\right)\right)$ in the output layer, where *J* is the number of the output layer and *g*(·) represents the linear function. The error *E*_j_(*n*) is denoted as $c_{j}(n)-t_{J}^{j}(n)$ in each layer. The total error *E*(n) is denoted as $\sum_{j=1}^{J}E_{j}^{2}(n)/2$.

#### Probabilistic Neural Network

The PNN is used to classify based on the Bayesian decision theory (Khan et al., 2015). PNN has the advantages of short duration, and the basis function has a little influence on the classification result.

Figure 1B shows the structure of the PNN. *x* represents the neuron of input layer. The neuron *φ*_ij_ in the hidden layer is denoted as $\exp[-(s-s_{ij}){\left(s-s_{ij}\right)}^{T}/\sigma^{2}]/{\left(2\pi \right)}^{\frac{1}{2}}\sigma^{d}$, where *s*_ij_ represents the *jth* core of *ith* sample, *σ* represents the smoothing factor and *d* represents the sample dimension. *ν*_i_ represents the relationship of the input and output sample in the hidden layer and is denoted as $\sum_{j=1}^{L}\varphi_{ij}/L$, where *L* represents the neuron number in the summation layer. *t* is denoted as *argmax* (*ν*_i_) which represents the relationship of the input and output sample in the output layer.

#### Competition Neural Network

In the competition NN, the output neurons compete with each other at the same time and only a winning neuron is finally selected. The learning rule is developed from the inner star rule.

Figure 1C shows the structure of the inner star model. *p*_i_ represents the neuron of input layer. *w*_i_ represents the connection weight. The output neuron *S* is denoted as *X*_w_ in the core layer. The adjustable weight Δ*w*_i_ is denoted as η(*p*_i_ − *w*_i_) *S*. *η* represents the learning rate.

#### Learning Vector Quantization Neural Network

The learning vector quantization (LVQ) NN was proposed by Kohonen (Hung et al., 2011). LVQ originated from the competition NN and each sample has its corresponding classified label.

Figure 1D shows the structure of LVQ network. *x* represents the neuron of input layer and *N* is the number of neuron. The first and second neurons correspond to the output label of *Y*_1_ and the third neuron corresponds to the output label of *Y*_2_ in the competition layer. *p*_i_ represents the *ith* input sample. *w*_1_ represents the weight between the input layer and the competition layer. The output *b* is denoted as *p*_i_*w*_1_.

#### Elman Neural Network

The Elman NN has the function of the local memory and feedback which helps to deal with the vary time series, thus this type of NN has high stability. Specifically, the memory function is reflected in the connection layer remembering the output of the layer hidden in the previous step (Wang et al., 2014).

Figure 1E shows the structure of Elman NN. *e*_i_(*f*) represents the *ith* input vector of the input layer at time *f*. The output at *f* time *q*_a_(*f*) in the connection layer is denoted as α*q*(*f* − 1), where α represents the delay at time *f* − 1. *q*(*f*) represents the output of hidden layer. *S*(*f*) represents the output of the output layer. *W*^1^ represents the weight between the connection layer and the hidden layer. *W*^2^ represents the weight between the input layer and the hidden layer. *W*^3^ represents the weight between the hidden layer and the input layer. The error * M* is denoted as [*S*_d_(*f*) − *S*(*f*)]^T^[*S*_d_(*f*) − *S*(*f*)]/2, where *S*_d_ and *S* represents the output and the actual output, respectively.

### The Application of Graph Theory

The human brain could be denoted by a complex network. Graph theory belonging to a branch of mathematics is used for analyzing the complex system. Therefore, the human brain network could be analyzed by graph theory. Graph theory has two important elements: nodes and edges.

The brain of each subject is divided into 90 regions (45 in each hemisphere) using anatomical automatic labeling (AAL) template (Plitt et al., 2015), which is regarded as the node of the brain network. The average time series of all voxels in a region are regarded as the time series of the region. The time series of two separated brain regions could be transformed into the Pearson correlation coefficient which forms a features matrix, and then the 90 diagonal elements are removed. These Pearson correlation coefficients are taken as the edge of the brain network. Thus there are 4005 (90 * 89/2) weighted edges. Then we used the absolute value of the correlation coefficient and set a fit threshold for the feature matrix to obtain an adjacency matrix. The threshold equals to 0.25 in this article.

The functional connectivity is usually selected as features between two brain regions (Plitt et al., 2015). There are also other indicators in graph theory analysis that could be selected as features such as the degree, clustering coefficient, shortest path and local efficiency of brain regions.

The degree of node represents the number of the directly linked edges. Shortest path is used for measuring the shortest path from a node to another node. The local efficiency reflects the capability of local information communication between one node and its neighbor nodes. It is assumed that *N*_i_ represents the degree of node *i*, *d*_ij_ represents the distance of node* i* and node* j*, *V*_i_ represents a node set in which all nodes directly connected to the node *i*. The local efficiency of node *i* is measured as $E(i)=\frac{1}{N_{i}(N_{i}-1)}\sum_{i\neq j\in V_{i}}\frac{1}{d_{ij}}$. Clustering coefficient reflects the degree of local cohesion between a node and its neighbor nodes. The clustering coefficient of node *i* measures as $C_{i}=\frac{e}{C_{N_{i}}^{2}}=\frac{2e}{N_{i}(N_{i}-1)}$, where *e* is the sum of adjacent edges. The number of degree, shortest path, clustering coefficient and local efficiency in each subject’s brain is 90, 4005, 90 and 90, respectively. Then the four indicators are integrated as the sample features of subsequent experiments.

### The Random Neural Network Cluster

#### The Design of the Random Neural Network Cluster

As a single NN has the advantages of dealing with the imperfect data and solving the problem of complex nonlinear systems, it is usually used for classification. However, the classification performance is not high and unstable. In this article, the random NN cluster is proposed by combining multiple NNs and it is an effective method to improve classification performance. The design of the random NN cluster is generated by the following steps.

Given a full dataset *D* = (***X***_1_, *Y*_1_), (***X***_2_, *Y*_2_), …, (***X***_*N*_, *Y*_*N*_), it contains *N* samples. *Y*_i_ represents the *ith* class label. ***X***_i_ represents the *ith* input sample which includes *M* features and it could be expressed as ***X***_i_ = (*x*_i1_, *x*_i2_, …, *x*_iM_), where *x*_ij_ represents the *jth* feature of the *ith* sample.

**Figure 2:**
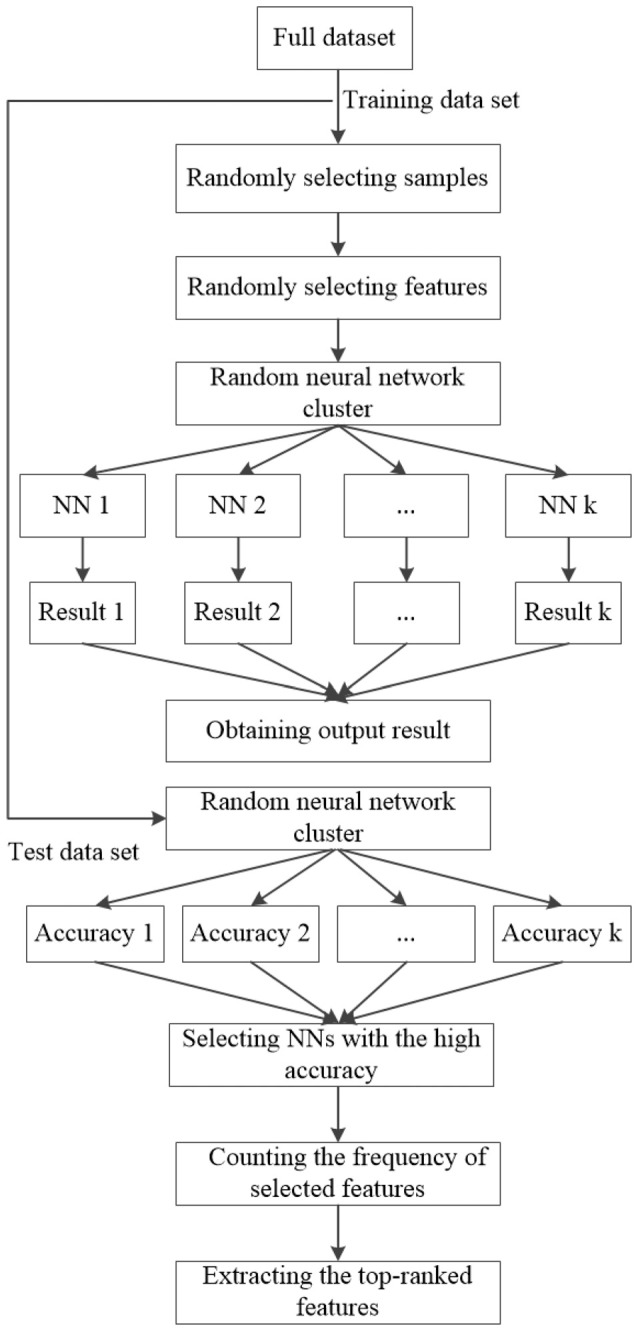
The formation of the random NN cluster.

The classification and feature extraction are carried out by using the random NN cluster. First, the full dataset *D* is divided into the training set *N*_1_ and the test set *N*_2_ and the proportion is 8:2. Second, *n* samples are randomly selected from (N_1_ (*N*_1_ ≫ *n*)) and *m* features are randomly selected from *M* (*M* ≫ *m*) to form a single NN. The process is repeated for *k* times. When there is a new sample entering into the random NN cluster, *k* NNs would have *k* classification results. Third, the majority of class labels are selected as the classification result of the random NN cluster. Fourth, the correctly predictive proportion of all the test samples *N*_2_ is regarded as the accuracy of the random NN cluster. Then, the NNs with the highest accuracy are selected out and the corresponding frequency of the selected features is counted. Finally, the features with top-ranked frequency are regarded as the significant features. The formation of the random NN cluster is shown in Figure 2.

#### The Classification of the Random Neural Network Cluster

In our experiment, there are 50 ASD patients and 42 TC. It is assumed that the class labels of TC and ASD patients are *h*_1_ and *h*_2_, respectively. As we use 90°, 90 clustering coefficients, 4005 shortest paths and 90 local efficiencies as the features, there are 4275 features for each subject. Thus, the sample feature could be defined as *X*_i_ *=* (*x*_i1_, *x*_i2_, …, *x*_i4275_), where *x*_i_ represents the *jth* feature of the *ith* subject. The classification method of the random NN cluster is described as the following.

First, 92 subjects are divided into a training set and a test set, and the proportion is 8:2. Thus the training set has 73 subjects and the test set has 19 subjects. Second, 70 subjects are randomly selected from 73 subjects and 120 features are randomly selected from 4275 features to establish a single NN, and this process is repeated for 1000 times to construct a random NN cluster. We calculate the accuracy of the NN using the toolbox of NN in the Matlab and the parameters of each NN are appropriate adjusted to get better classification results. Third, we apply five types of NNs (BP) NN, Elman NN, Probabilistic NN, LVQ NN and Competitive NN) to construct the five types of random NN clusters, which are the random BP NN cluster, the random Elman NN cluster, the random Probabilistic NN cluster, the random LVQ NN cluster and the random Competition NN cluster. In the five types of random NN clusters, the base classifier of the random NN cluster with highest accuracy is regarded as the best base classifier.

Finally, 19 samples enter into the random NN cluster, and 1000 NNs make decisions at the same time to obtain the classification result of each sample. The majority of class labels are regarded as the predictive label of each sample. When the predictive class label is the same as the real class label, the label is called as consistent label. The accuracy of the random NN cluster equals to the number of consistent label divided by 19.

In the 1000 NNs, not every NN contributes to the random NN cluster. Thus, it is important to find out the significant NNs which contribute greatly to the random NN cluster. In this article, we select the NNs from 1000 NNs whose accuracies are greater than 0.6 as the significant NNs.

#### Extracting Features From the Random Neural Network Cluster

As each NN has different characteristics, the selected features would make different contributions to NN and the random NN cluster. Therefore, it is necessary to select the significant features which could reflect the classification performance between the ASD patients and TC based on fMRI data. The process of extracting features is as follows.

First, the samples and features are randomly selected from the training set to construct the random NN cluster. Second, the samples of the test set enter into each NN of the random NN cluster to get the accuracies of 1000 NNs. Third, the NNs whose accuracies are greater than 0.6 are selected from 1000 NNs, and we call these NNs as the significant NNs. Fourth, we select the features of significant NNs from the total 4275 features, and these features are sorted in a descending order according to their frequencies. Next, the features with high frequency are considered as the significant features which could be used to distinguish between ASD patients and TC. Then, we select a part of significant features as the sample features to construct a random NN cluster and calculate their accuracies. Finally, the number of significant features corresponding to the random NN cluster with the highest accuracy is the optimal number.

After completing the features extraction in the whole brain, we use the significant features to find out the abnormal brain regions between ASD patients and TC. In order to estimate the abnormal degree of a brain region, the number of features which are related to the brain region is regarded as the criteria. If the brain region is not related to any significant feature, the weight of the brain region is 0. The greater the number of features is, the higher the abnormal degree is.

## Results

### The Performances of the Random Neural Network Cluster

**Figure 3:**
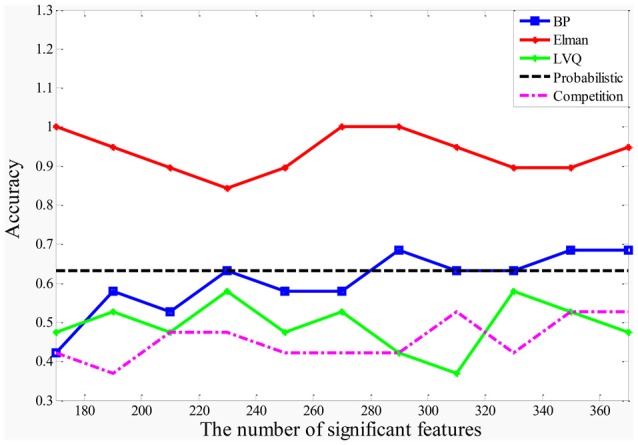
The accuracies of the five types of random NN clusters.

In this article, the five different types of NNs (BP NN, Elman NN, Probabilistic NN, LVQ NN and Competitive NN) are applied to construct the five types of random NN clusters. The classification performances of the five types of random NN clusters are shown in Figure 3. It is referred that the accuracies of the random Competition NN cluster and the accuracies of the random LVQ NN cluster are not high; the accuracies of the random Elman NN cluster fluctuate around 95%, even nearly reach to 100%; the accuracies of the random BP NN cluster and the random Probabilistic NN cluster are higher than the random Competition NN cluster and the random LVQ NN cluster. Thus, we finally select the Elman NN as the best base classifier and the subsequent results are acquired based on the random Elman NN cluster.

**Figure 4:**
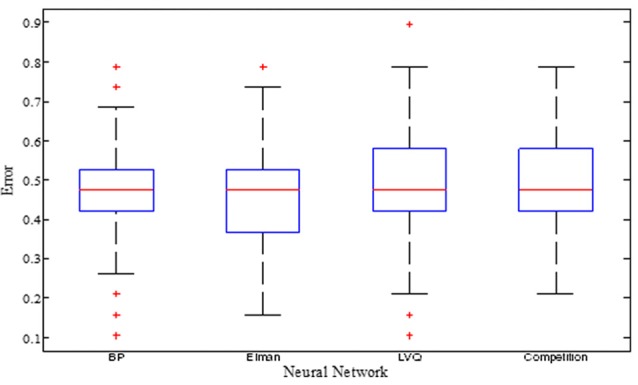
The accuracies of 1000 NNs in four types of random NN clusters.

The training errors and the test errors of the five types of random NN clusters are shown in Table 2. Figure 4 shows the accuracies of 1000 NNs in four types of random NN clusters. As the accuracies of 1000 NNs in the random probabilistic NN cluster are the same values, Figure 4 does not show the accuracies of probabilistic NNs. From the Table 2 and Figure 4 we could learn that the error is higher in a single NN and is lower in a random NN cluster.

**Table 2:** The errors of the five types of random neural network (NN) clusters.

Variables (Mean ± SD)	Training errors	Test errors
Random BP neural network cluster	0.60 ± 0.09	0.60 ± 0.08
Random probabilistic neural network cluster	0.63 ± 0.00	0.63 ± 0.00
Random Elman neural network cluster	0.93 ± 0.04	0.93 ± 0.05
Random LVQ neural network cluster	0.50 ± 0.06	0.49 ± 0.06
Random competition neural network cluster	0.46 ± 0.07	0.45 ± 0.05

In order to show the performance of the random NN cluster, we compared the non-NN variant of the classifiers (the support vector machine (SVM) and the decision-making tree) with the NN. When the decision-making tree is made as the classifier, the highest classification accuracy is 87%. When the SVM is made as the classifier, the highest classification accuracy is 84%. These are lower than the highest classification accuracy of the random NN cluster.

In our experimental results, when the number of the classifier is 270 which could be discussed in the following part, the accuracy of the random Elman NN cluster is the highest. Therefore, we fix the number of base classifiers on 270. Then, we repeat the experiments for 50 times, and obtain the results of their classifiers’ accuracies. The differences between the base classifier of Elman NN and the base classifier of the decision-making tree are tested by the two-sample *t*-test. *P* value is close to 0.015, which refers that these two groups have statistical significance. The differences between the base classifier of Elman NN and the base classifier of the SVM are tested by the two-sample *t*-test. *P* value equals to 0.000, which refers that these two groups have statistical significance. Table 3 shows the result of statistical significance between the three methods.

**Table 3:** The result of statistical significance between the three methods.

Base classifier (Mean ± SD)	SVM	Elman NN	Decision tree	*P* value
Accuracy (%)	0.773 ± 0.034	0.847 ± 0.032	0.834 ± 0.016	0.000^a^/0.015^b^

*^a^the p value of the two-sample t-test between SVM and NN. ^b^the p value of the two-sample t-test between decision tree and NN*.

### The Significant Neural Networks and Features

When the samples of the test set enter into the random Elman NN cluster, we could obtain the accuracies of 1000 Elman NNs. We select the Elman NN whose accuracy is greater than 0.6 as the significant Elman NNs, and the result indicates that the number of significant Elman NNs is 270. These significant Elman NNs make great contributions to distinguish between ASD and TC in the random Elman NN cluster.

After determining the significant Elman NNs, we could select significant features with higher accuracy from these NNs. In order to determine the optimal number of the significant features, we make the accuracy of the random Elman NN cluster as the criteria. Figure 3 shows the accuracies of the random Elman NN cluster with different numbers of significant features. It is referred that when the number of significant features is 170, 260, 270 and 280, the accuracies of the random Elman NN cluster fluctuate around 95%, even nearly reach to 100%. But when the number of features is 170, the accuracies of the random Elman NN cluster are not stable. We choose the 270 as the optimal number of significant features because the accuracies of the random Elman NN cluster are highest and stable.

### The Abnormal Brain Regions

In this article, we focus on the brain regions of which the weights are higher than 11. They are the Supp_Motor_Area (SMA), the Cingulum_Mid (DCG), the Fusiform (FFG), the Insula (INS), the Frontal_Inf_Oper (IFGoperc), the Cingulum_Post (PCG), the Calcarine (CAL), the Occipital_Sup (SOG).

**Figure 5:**
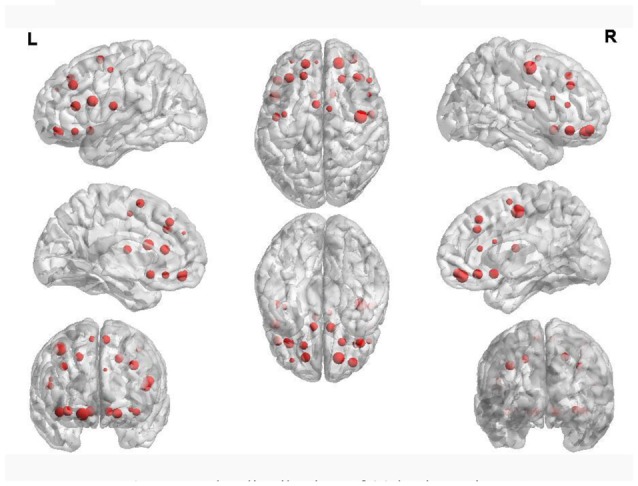
The distribution of 90 brain regions.

Table 4 shows the regions whose weights are higher than 11 and their corresponding volumes. Figure 5 shows the distribution of 90 brain regions using Brain-NetViewer^3^. The red nodes indicate the brain regions, and the size of the nodes indicates the abnormal degree of the brain regions. The greater the node is, the higher the abnormal degree is.

**Table 4:** The regions with higher weight.

Regions	The volume of region	Weight
SMA.R	[9 062]	18
DCG.R	[8 −9 40]	15
FFG.L	[−31 40 −20]	
INS.L	[−3 5 73]	14
IFGoperc.R	[5015 21]	13
INS.R	[3962]		
DCG.L	[−5 −15 42]	12
PCG.R	[7 −42 22]	
CAL.L	[−7 −79 6]	
SOG.L	[−17 84 28]	
PreCG.L	[−39–651]	11
SFGdor.R	[22 31 44]	
MFG.L	[−33 33 35]	
ROL.L	[−47 −8 14]	
SFGmed.R	[9 51 30]	
PCG.L	[−5 43 25]	
PCL.L	[−8 25 70]	

## Discussion

### Classification Performance

In recent years, there are some researchers trying to classify and diagnose ASD patients from TC. Wang et al. (2012) used fMRI data to classify ASD patients and TC with the classification sensitivity of 82.8% and the specificity of 82.8%. Ecker et al. (2010) applied SVM to classify ASD patients and TC and the sensitivity and specificity of classification was 90% and 80% respectively. Uddin et al. (2013) employed functional connectivity to classify ASD patients and TC, and the classification accuracy was 78%. As the classification accuracy is not high in most existing studies, the random NN cluster is proposed to improve the classification performance in the diagnosis of ASD. In this article, five different NNs were applied to construct the five types of random NN clusters. The highest accuracy of the random BP NN cluster and the random Probabilistic NN cluster are 68.4% and 63.2% respectively. The highest accuracy of the random LVQ NN cluster and the accuracy of random Competitive NN cluster are only 57.8% and 52.6%. We ultimately selected the Elman NN as the best base classifier and the high accuracy of the random Elman NN cluster nearly reaches to 100%. The experimental results show that the performance of the random NN cluster is very good.

The Elman NN is able to deal with the dynamic data, thus it is suitable for the fMRI data which changes in a period of time. In general, the random Elman NN cluster could be applied to the rapid and accurate detection of the abnormal brain regions in ASD patients.

### Additional Discussion of the Random Neural Network Cluster

In this part, we discuss the additional issues including the parameters, complexity, errors, weight and the overfitting of the random NN cluster.

In the random NN cluster, the parameters are decided by the accuracy of the random NN cluster. Besides, the importance of parameters in bad classifier could be reduced by the randomness of the random NN cluster. In a single NN, the parameters are adaptive and they are under the control of NN toolbox. Generally, after a series of strict process of parameters selection, the performance of the random NN cluster improves.

The random NN cluster is complicated which is reflected in the following two points. On the one hand, the process of constructing a random NN cluster is complex because the number of base classifiers is 1000. On the other hand, the process of finding the optimal number of base classifiers is complex because we need to select the optimal number of base classifiers based on the accuracy of the random NN cluster constructed by different number of base classifiers.

In terms of the weight, there are two kinds of weights in our method. When the accuracy of the random NN cluster is calculated, the percentage of voting for each base classifier (NN) is the same. In the interior structure of a single NN, the weight is set by the NN toolbox.

The subjects have been divided into a training set and a test set. The training set is used for building the random NN cluster, and the test set is used for testing the performance of the random NN cluster. Our experimental results show that the random NN cluster works well on the test set, thereby there is no overfitting. In addition, a random NN cluster was composed of many NNs and each NN is unique due to the random samples and random features, which also ensures that there is no overfitting.

### Analysis of the Significant Neural Networks and Features

To classify ASD patients from TC correctly, we selected the significant NNs and features. In this article, we used five different types of NNs to construct five types of random NN clusters and the process of establishing a single NN repeated for 1000 times to construct a random NN cluster. The accuracies of the five types of random NN clusters were compared, and then the corresponding NN in the random NN cluster with the highest accuracy was selected as the best base classifier. When the number of NNs is large, it is more difficult to calculate. But the classification result is more consistent with the actual result in this situation. Thus, it is important to select an appropriate number of NNs. In this article, we used 1000 NNs to construct the random NN cluster. As each NN has its own characteristics, the NNs make different contributions to the random NN cluster. The greater the accuracy of NN is, the higher the contribution is. If the accuracy is more than 0.5, the classification is good in machine learning (Krishnan and Westhead, 2003). The accuracy of threshold is generally artificially set, and we selected the Elman NNs whose accuracies were greater than 0.6 as the significant Elman NNs. To select these significant features, we firstly made a preliminary filtration from the 4275 sample features to select a part of features. These features were sorted in a descending order according to their frequencies and the features with higher frequency are considered as the significant features. It is the above process of feature extraction that makes our method different from other methods. Our method is able to make full use of all features and select out appropriate significant features at higher speed.

### Analysis of the Brain Regions With the Greater Weight

In this article, the random NN cluster has been applied to classify ASD patients from TC and find out the abnormal brain regions. Some abnormal brain regions were found out corresponding to AAL template in ASD patients such as the supplementary motor area (SMA.R), the median cingulate and paracingulate gyri (DCG), the fusiform gyrus (FFG.L), the insula (INS), the inferior frontal gyrus (IFGoperc.R), the posterior cingulate gyrus (PCG), the calcarine fissure and surrounding cortex (CAL.L), the superior occipital gyrus (SOG.L), the precentral gyrus (PreCG.L), the superior frontal gyrus (SFGdor.R), the middle frontal gyrus (MFG.L), the rolandic operculum (ROL.L), the medial of superior frontal gyrus (SFGmed.R) and the paracentral lobule (PCG.L). In many studies, some abnormal regions were found out in ASD patients. For instance, Murdaugh et al. (2012) concluded that ASD patients had less deactivation in DMN regions including medial prefrontal cortex, anterior cingulate cortex and posterior cingulate gyrus. Itahashi et al. (2015) discovered local functional disruptions in the right superior frontal gyrus and middle frontal gyrus in ASD patients. Choi et al. (2015) found out abnormal regions of ASD patients in the right dorsolateral prefrontal cortex, the right parietal lobe, the right orbitofrontal cortex and the superior temporal gyrus. Subbaraju et al. (2017) concluded that the prefrontal cortex, the posterior and medial portions were abnormal in ASD patients.

Our experimental results are consistent with these findings. In this article, we focused on some abnormal regions which had larger frequency such as the supplementary motor area, the cingulate gyrus, the FG and the INS.

#### The Supplementary Motor Area (SMA.R)

The SMA.R had the greatest frequency in the abnormal brain regions in ASD patients. It is referred that the SMA.R makes a great contribution to classify ASD and TC in the random Elman NN cluster. The SMA is linked to the function of movement observation, preparation and execution (Enticott et al., 2009). It is responsible for planning and executing motor tasks (Hupfeld et al., 2017).

Our experimental results are consistent with many previous studies. Chen et al. (2015) found that intrinsic functional connectivity was related to the somatosensory default mode and visual regions in ASD patients. Kestemont et al. (2016) explored that there were more activation differences between ASD patients and TC concentrating in the SMA, the left precentral gyrus and so on. Fournier et al. (2010) observed that the motor dysfunction in SMA could be a feature of diagnosing ASD. Ewen et al. (2016) detected the abnormal regions of ASD patients locating in the motor network which includes the SMA.

The abnormal SMA may lead to the physical movement deficits in ASD patients. The above results reveal that SMA may be a clinical and pervasive feature to diagnose ASD in the future.

#### The Cingulate Gyrus

The cingulate gyrus had the higher frequency in the abnormal brain regions. It is referred that the cingulate gyrus makes a great contribution to classify ASD and TC in the random Elman NN cluster. The cingulate gyrus is associated with the neurocognitive function (Calabrese et al., 2008), the somatosensory function (Nair et al., 2015) and the behaviors and cognitive processes (Apps et al., 2016).

Our experimental results are consistent with many previous studies. Cascio et al. (2014) discovered that the INS and the anterior cingulate cortex were abnormal regions in ASD patients. Thakkar et al. (2008) concluded that the abnormalities of the anterior cingulate cortex in ASD could make contributions to repetitive behavior. Apps et al. (2016) found out the abnormal regions such as the left orbitofrontal cortex and left posterior cingulate gyrus in ASD patients.

The abnormal cingulate gyrus may lead to the cognitive processes deficits in ASD patients. The above results reveal that the cingulate gyrus may be a clinical and pervasive feature to diagnose ASD in the future.

#### The Fusiform Gyrus (FG)

The FG had the higher frequency in the abnormal brain regions in ASD patients. It is referred that the FG makes a great contribution to classify ASD patients and TC in the random Elman NN cluster. The FG is associated with the social-emotional and face recognition (Oblak et al., 2010; Hernandez et al., 2015).

Our experimental results are consistent with many previous studies. Yucel et al. (2014) found the difference between ASD patients and TC involved in the amygdala and the FG. Apps et al. (2016) discovered that the amygdala and the FG were abnormal in ASD patients. Kaiser et al. (2016) found out some abnormal regions such as the FG.R, the right amygdala and the bilateral ventrolateral prefrontal cortex in ASD patients.

The abnormal FG may lead to the face recognition deficits in ASD patients. This founding reveal that the FG may be regarded as a new biomarker to further test the disease of ASD and provide convenience for clinical diagnosis of ASD.

#### The Insula (INS)

The INS had a relatively higher frequency in the abnormal regions, thus it is referred that the INS makes a great contribution to classify ASD and TC in the random Elman NN cluster. The INS are relevant to the cognition mechanism (Uddin and Menon, 2009).

Our experimental results are consistent with many previous studies. Murdaugh et al. (2012) found out some abnormal regions of ASD patients including the posterior cingulate gyrus, the INS and the SMA. Plitt et al. (2015) explored that the inferior frontal gyrus and the INS were abnormal in ASD patients. Keehn et al. (2016) detected that the occipital cortex, the dorsolateral prefrontal cortex and the INS were abnormal in ASD patients.

The abnormal INS may lead to the simulation mechanism deficits in ASD patients. These results reveal that the INS may be regarded as a new biomarker to further test the disease of ASD and provide convenience for clinical diagnosis of ASD.

In this article, the random NN cluster was proposed to classify ASD patients from TC and found out the abnormal brain regions in ASD patients based on the fMRI data. The new method has some advantages. On the one hand, we selected the random Elman NN cluster from five types of random NN clusters and its highest accuracy even could nearly reach to 100%. On the other hand, we used the random Elman NN cluster could find the significant features which are the most differences between ASD patients and TC. Therefore, the random NN cluster might be an appropriate approach for diagnosing ASD. There are some limitations. First, our experimental sample size is not large. In this article, 92 participants were selected from ABIDE which was the maximum number of samples that we could obtain. In the future studies, the new method can be applied to the larger sample size. Second, this article integrated the four indicators as the features of subjects. In the future studies, we could also integrate other indicators as features.

## Ethics Statement

This study was carried out in accordance with the recommendations of National Institute of Aging-Alzheimer’s Association (NIA-AA) workgroup guidelines, Institutional Review Board (IRB). The study was approved by Institutional Review Board (IRB) of each participating site, including the Banner Alzheimer’s Institute, and was conducted in accordance with Federal Regulations, the Internal Conference on Harmonization (ICH) and Good Clinical Practices (GCP).

## Author Contributions

X-aB proposed the design of the work and revised it critically for important intellectual content. QSun and QJ carried out the experiment for the work and drafted part of the work. YL, QShu and JD collected, interpreted the data and drafted part of the work. All the authors approved the final version to be published and agreed to be accountable for all aspects of the work in ensuring that questions related to the accuracy or integrity of any part of the work are appropriately investigated and resolved.

## Conflict of Interest Statement

The authors declare that the research was conducted in the absence of any commercial or financial relationships that could be construed as a potential conflict of interest.

## Footnotes

^1^http://fcon_1000.projects.nitrc.org/indi/abide/

^2^http://d.rnet.co/DPABI/DPABI_V2.3_170105.zip

^3^http://www.nitrc.org/projects/bnv/
